# Reprogramming the Tumor Microenvironment in Head and Neck Squamous Cell Carcinoma: Therapeutic Targets and Innovations

**DOI:** 10.32604/or.2025.068395

**Published:** 2025-10-22

**Authors:** Bruno Špiljak, Bojan Poposki, Stjepanka Lešić

**Affiliations:** 1 Department of Oral Medicine, University of Zagreb School of Dental Medicine, Zagreb, 10000, Croatia; 2 Department of Oral and Periodontal Diseases, Faculty of Dentistry-Skopje, Ss. Cyril and Methodius University in Skopje, Skopje, 1000, North Macedonia; 3 Department of Dental Medicine, Faculty of Dental Medicine and Health Osijek, J. J. Strossmayer University of Osijek, Osijek, 31000, Croatia

**Keywords:** Head and neck squamous cell carcinoma, tumor microenvironment, cancer-associated fibroblasts, immunotherapy, translational oncology

## Abstract

Head and neck squamous cell carcinoma (HNSCC) is an aggressive cancer with high recurrence rates and prevalent resistance to therapeutic interventions. Tumor behavior is largely dependent on the tumor microenvironment (TME) that includes immune cells, stromal components, cancer-associated fibroblasts (CAFs), the extracellular matrix (ECM), and an associated cytokine network. In this review, we examine principal mechanisms of the tumorigenic transformation, encompassing immune checkpoint disruption, therapy resistance mediated through CAFs, the contribution of hypoxic niches, and several metabolic dependencies that hold potential as future targets. Novel therapeutics developed and/or repurposed, such as immune checkpoint inhibitors (ICIs), TME modulation therapeutics, CAF reprogramming, hypoxia targeting agents, and ECM remodeling, aim to overcome TME-mediated resistance. We also examine the rationale and progress of integrating TME-targeted therapies with other treatment modalities. By identifying actionable, molecular targets within the HNSCC TME, this review presents a translational perspective for implementing TME modulation in personalized treatment. The challenges comprise TME heterogeneity, a paucity of predictive biomarkers, and a translational gap between pre-clinical and clinical practice. Future studies must be aimed at proper stratification of patients, optimization of combination treatment, and cost-effectiveness analysis of TME-modifying therapies to enable personalized medicine in HNSCC treatment.

## 1 Introduction

Head and neck squamous cell carcinoma (HNSCC) remains a major global health burden, ranking as the sixth most common cancer worldwide and accounting for more than 600,000 new cases and over 350,000 deaths annually [1]. Despite advances in surgical techniques, radiation therapy, and chemotherapy protocols, the overall five-year survival rate for HNSCC has only modestly improved over the past few decades, lingering around 50%–60% depending on the tumor stage and anatomical site [2]. This stagnation in outcomes is largely attributable to the high rates of locoregional recurrence, distant metastasis, and intrinsic or acquired resistance to conventional therapies. Traditional therapeutic approaches mainly targeted cancer cells directly, based on the implicit assumption that eradicating the tumor depended primarily on eliminating malignant cells [3]. However, emerging evidence underscores the critical importance of the tumor microenvironment (TME) in governing cancer behavior, influencing not only tumor growth and dissemination but also immune evasion and therapy resistance [4,5]. In HNSCC, the TME constitutes a particularly dynamic and immunosuppressive milieu, characterized by the infiltration of regulatory T cells (Tregs), myeloid-derived suppressor cells (MDSCs), tumor-associated macrophages (TAMs), dense networks of cancer-associated fibroblasts (CAFs), altered extracellular matrix (ECM) architecture, hypoxia-driven signaling, and complex cytokine and chemokine networks [6–8]. The functional heterogeneity and plasticity of the TME components not only facilitate tumor progression but also actively thwart anti-tumor immune responses and blunt the efficacy of immunotherapy and cytotoxic agents. For instance, overexpression of immune checkpoints such as programmed death-ligand 1 (PD-L1), recruitment of immunosuppressive cell subsets, metabolic reprogramming, and the development of hypoxic niches collectively establish formidable barriers to durable therapeutic responses [9,10]. In this context, the TME is now recognized not merely as a passive scaffold but as an active co-conspirator in HNSCC pathogenesis and therapy resistance. The limitations of monotherapies—whether immune checkpoint inhibitors (ICIs), chemotherapy, or targeted agents, have driven research into combinatorial strategies that incorporate TME modulation as a cornerstone of treatment [10–12]. By reprogramming the immunosuppressive, fibrotic, and hypoxic features of the TME, researchers increasingly believe that current therapies could become more effective, durable, and accessible to a broader patient population [13]. In this review, we synthesize the current understanding of the HNSCC TME, highlighting key cellular and molecular drivers of immunosuppression and resistance, and exploring emerging therapeutic strategies designed to reprogram or disrupt the TME to favor anti-tumor immunity and treatment responsiveness. We focus on recent insights into immune checkpoint dysregulation, the role of CAFs and stromal remodeling, hypoxia-driven metabolic vulnerabilities, and novel drug delivery strategies tailored to overcome TME-related barriers. Furthermore, we examine ongoing clinical trials and translational perspectives that seek to integrate TME-targeted approaches into standard-of-care regimens, ultimately advocating for a multidisciplinary, biomarker-informed framework for the personalized management of HNSCC.

## 2 The Tumor Microenvironment in Head and Neck Squamous Cell Carcinoma

**Figure 1:**
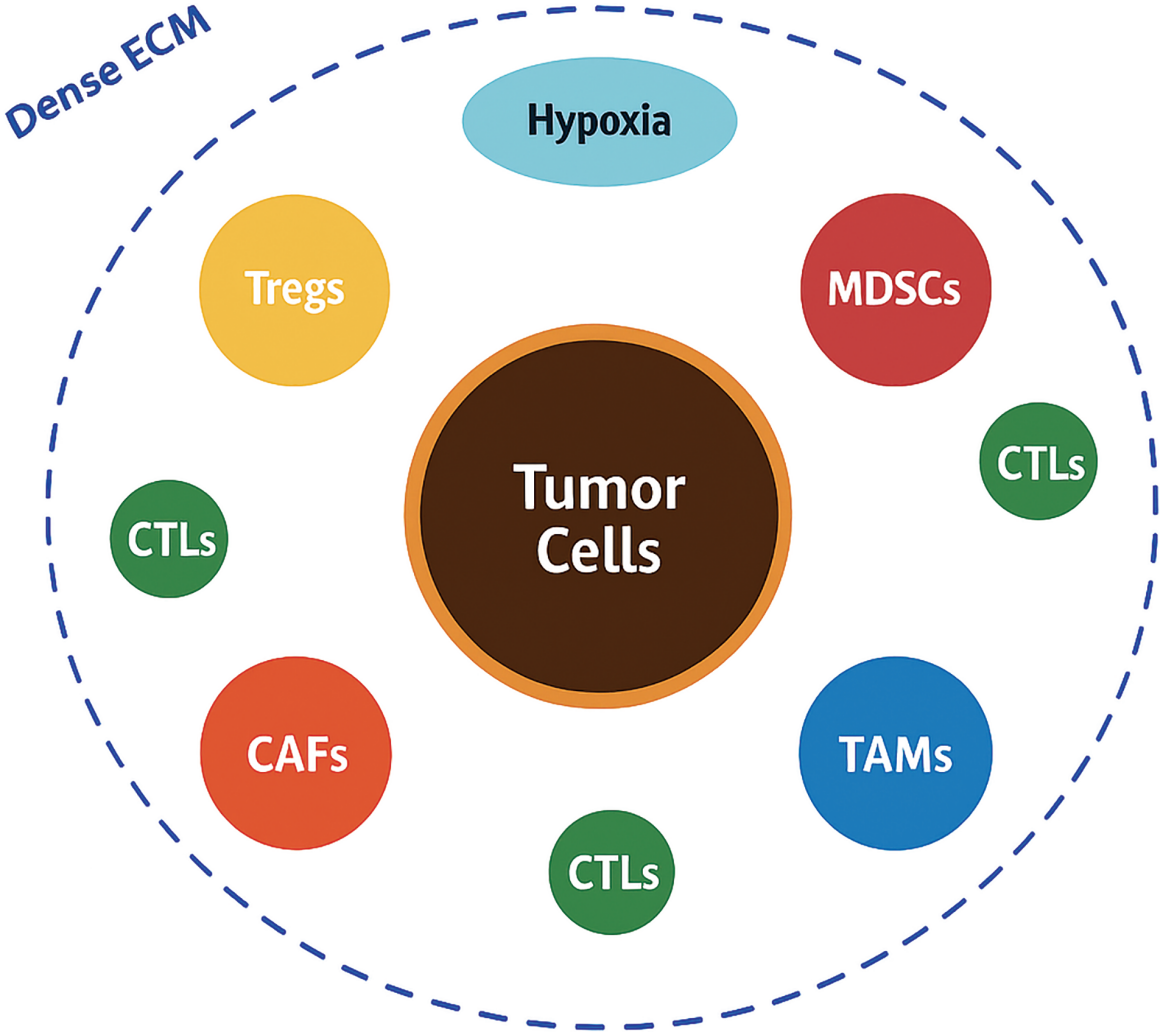
Schematic overview of the tumor microenvironment in head and neck squamous cell carcinoma. Tumor cells occupy the center and are encircled by various immunosuppressive and stromal components, including regulatory T cells (Tregs), myeloid-derived suppressor cells (MDSCs), cancer-associated fibroblasts (CAFs), and tumor-associated macrophages (TAMs). A dense extracellular matrix (ECM) barrier, shown as a thick collagenous layer, physically and biochemically isolates the tumor, while hypoxic regions (in blue) further create an immunosuppressive niche. A few cytotoxic T lymphocytes (CTLs) appear trapped at the periphery, illustrating the immune-excluded phenotype typical of head and neck squamous cell carcinoma (HNSCC) (Original schematic created using BioRender.com, BioRender, Toronto, ON, Canada)

The TME of HNSCC is a highly dynamic and heterogeneous ecosystem composed of cellular and non-cellular components that interact closely with malignant cells to shape tumor progression, immune evasion, metastasis, and therapeutic resistance [14] (Fig. 1).

Unlike the traditional view of cancer as a purely cell-autonomous process, researchers now recognize that the bidirectional communication between tumor cells and their surrounding microenvironment plays a pivotal role in dictating disease behavior and clinical outcomes [6,15]. Among the cellular constituents, immune cells form a critical component of the HNSCC TME. Despite the presence of tumor-infiltrating lymphocytes (TILs), the immune contexture is predominantly immunosuppressive. Tregs, characterized by the expression of Forkhead Box P3 (FOXP3), accumulate within the tumor bed and secrete immunosuppressive cytokines such as transforming growth factor beta (TGF-β) and interleukin-10 (IL-10), thereby inhibiting effective cytotoxic T lymphocyte (CTL) responses [5,16]. In parallel, MDSCs expand both systemically and within the tumor, producing reactive oxygen species, nitric oxide, and arginase-1, which collectively impair T-cell proliferation and antigen presentation [17]. TAMs, often polarized towards an M2-like phenotype, further contribute to immunosuppression by secreting vascular endothelial growth factor (VEGF), IL-10, and promoting tissue remodeling and angiogenesis [18,19]. CAFs are another indispensable and highly influential cellular component of the HNSCC TME. CAFs secrete a plethora of growth factors, cytokines, and ECM components that drive tumor proliferation, invasion, and metastasis. They are key mediators of ECM remodeling, producing dense collagen networks, fibronectin, and tenascin-C, which create a rigid and desmoplastic stroma that acts as both a physical and biochemical barrier to immune infiltration and therapeutic penetration [20–22]. Moreover, CAFs actively modulate immune responses by expressing immunomodulatory molecules such as PD-L1, and by secreting chemokines like C-X-C Motif Chemokine Ligand 12 (CXCL12) which exclude cytotoxic immune cells from the tumor core [23–25]. The non-cellular components of the TME, particularly the ECM and soluble factors, also play critical roles. The ECM not only provides structural support but also influences cellular behavior through mechanotransduction and by serving as a reservoir for growth factors. Dysregulated ECM composition and stiffness promote epithelial-mesenchymal transition (EMT), facilitate metastatic dissemination, and enhance therapeutic resistance [4,26,27]. An important hallmark of the HNSCC microenvironment is hypoxia, resulting from abnormal vasculature and rapid tumor growth. Hypoxia stabilizes hypoxia-inducible factors (HIFs), which drive angiogenesis, metabolic reprogramming (favoring glycolysis and lactate production), immune escape, and resistance to radiotherapy [28,29]. The accumulation of metabolic byproducts such as lactate acidifies the microenvironment, further impairing effector immune cells and favoring regulatory populations. TME heterogeneity represents a significant clinical challenge. Spatial and temporal differences in immune infiltration, CAF activation states, vascularization, and hypoxic gradients create distinct micro-niches within tumors, each with variable sensitivity to treatments [6,30,31]. Such heterogeneity underlies the variability in patient responses to therapy and complicates the identification of reliable predictive biomarkers. From a prognostic standpoint, several studies have demonstrated that a “hot” immune microenvironment, characterized by abundant activated CTLs and low levels of suppressive elements, correlates with better outcomes, whereas a “cold” TME, dominated by immunosuppressive cells and desmoplastic stroma, predicts poorer prognosis and resistance to immunotherapy [30,32]. Understanding the intricate composition and functional dynamics of the HNSCC TME is therefore essential for developing effective therapeutic strategies. Targeting individual components of the TME, whether through immune checkpoint blockade, stromal modulation, hypoxia targeting, or metabolic reprogramming, has the potential to recondition the microenvironment, restore anti tumor immunity, and enhance treatment efficacy (Table 1). In the following sections, we explore these strategies, highlighting the most promising approaches to therapeutically reprogram the hostile microenvironment of HNSCC.

**Table 1:** Key components of the head and neck squamous cell carcinoma tumor microenvironment (an original table reproduced from the current literature data)

Component	Subtype/Example	Main roles in TME
**Immune cells**	Tregs, MDSCs, TAMs	Immune suppression, promotion of tumor growth, resistance to immunotherapy [5,16–19]
**Stromal cells**	CAFs	ECM remodeling, immune exclusion, secretion of pro-tumorigenic cytokines [20–24]
**ECM**	Collagen, Fibronectin, Tenascin-C	Physical barrier to drug delivery, supports invasion and metastasis [4,26,27]
**Hypoxia**	HIF-1α stabilization, hypoxic niches	Angiogenesis, immune escape, metabolic reprogramming [28–30]

Note: Tregs, Regulatory T Cells; MDSCs, Myeloid-Derived Suppressor Cells; TAMs, Tumor-Associated Macrophages; CAFs, Cancer-Associated Fibroblasts; ECM, Extracellular Matrix; HIF-1α, Hypoxia-Inducible Factor 1-Alpha

## 3 Immune Checkpoint Dysregulation in Head and Neck Squamous Cell Carcinoma

**Figure 2:**
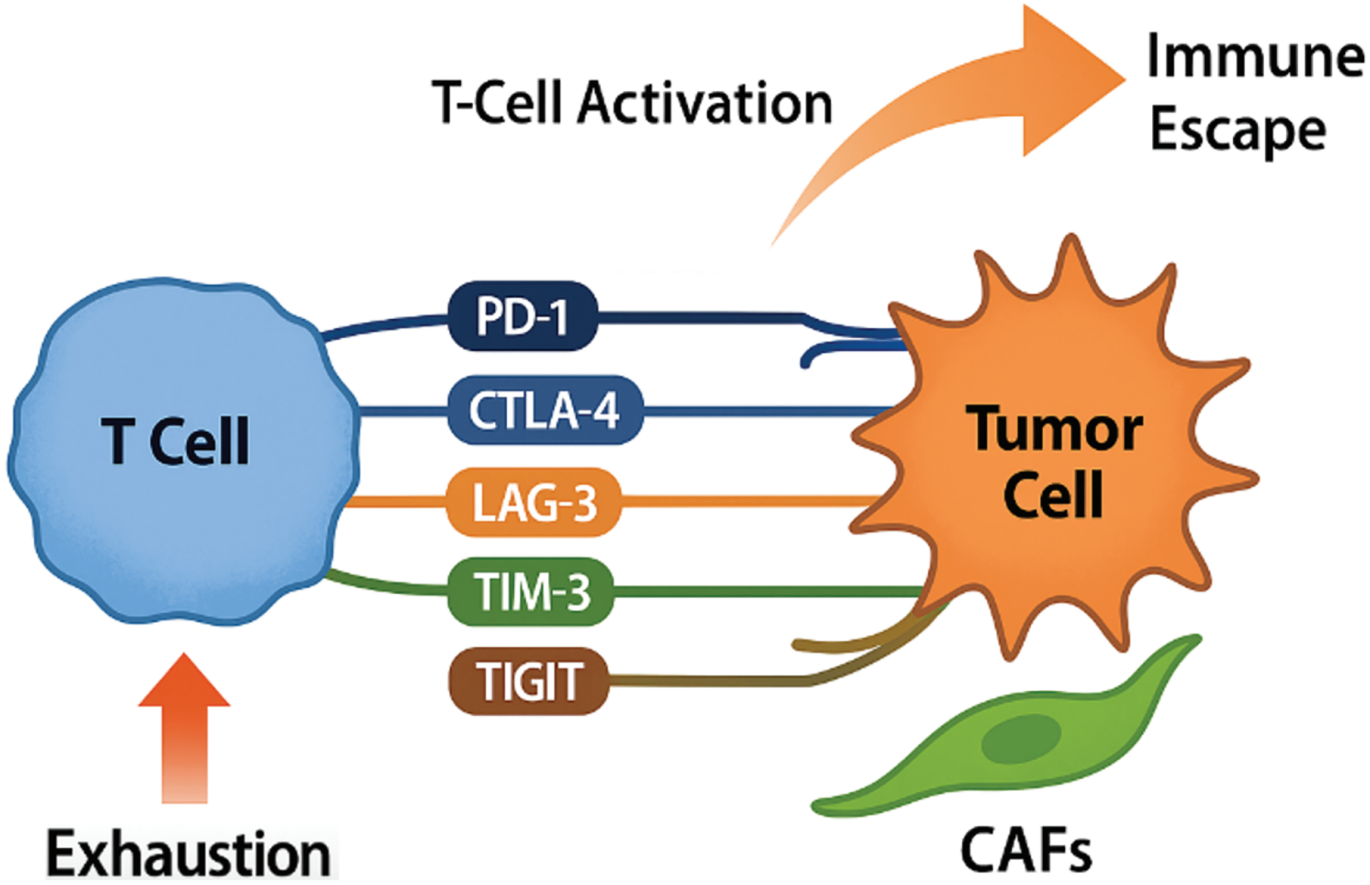
Mechanisms of Immune checkpoint dysregulation in head and neck squamous cell carcinoma. Upon T-cell activation, there is an upregulation of multiple inhibitory immune checkpoints including programmed cell death protein 1 (PD-1), cytotoxic T-lymphocyte-associated antigen 4 (CTLA-4), lymphocyte activation gene 3 (LAG-3), T-cell immunoglobulin and mucin domain-containing protein 3 (TIM-3), and T-cell immunoreceptor with Ig and ITIM domains (TIGIT). These receptors interact with their corresponding ligands, primarily expressed by tumor cells and im-mune-infiltrating cells, most notably programmed death-ligand 1 (PD-L1). This interaction leads to T-cell exhaustion, characterized by reduced effector function and proliferative capacity, ultimately facilitating immune escape, impaired tumor immunosurveillance, and continued progression of head and neck squamous cell carcinoma (HNSCC) (Original scheme based on current literature data created using BioRender.com BioRender, Toronto, ON, Canada)

Immune evasion is a hallmark of cancer, and in HNSCC, dysregulation of immune checkpoint pathways serves as a central mechanism by which tumors escape immune surveillance [33,34] (Fig. 2).

The TME of HNSCC typically shows high expression of inhibitory molecules such as PD-L1 on both tumor cells and immune-infiltrating cells, along with the upregulation of co-inhibitory receptors including programmed death-1 (PD-1) and cytotoxic T-lymphocyte-associated protein 4 (CTLA-4) on T cells [35–38]. The PD-1/PD-L1 axis is particularly significant in HNSCC. PD-L1 binding to PD-1 inhibits T-cell receptor (TCR) signaling, leading to T-cell anergy, exhaustion, and apoptosis, effectively blunting anti-tumor immune responses [39,40]. Clinical trials that target this pathway have transformed HNSCC treatment paradigms. Notably, the phase III KEYNOTE-048 trial demonstrated that pembrolizumab, an anti-PD-1 antibody, improved overall survival compared to standard chemotherapy in patients with PD-L1-positivel recurrent or metastatic HNSCC [41]. Similarly, nivolumab produced durable responses and survival benefits in heavily pretreated populations, as demonstrated in the CheckMate 141 trial [35]. However, the overall response rates (ORRs) to PD-1/PD-L1 blockade remain modest, typically around 15%–20% in unselected patient populations [36,42–44]. The variability in response to ICIs highlights the need for predictive biomarkers to guide patient selection [45,46]. Among these, PD-L1 expression is the most commonly used in clinical practice but has several limitations [46]. Tumor mutational burden (TMB) has shown promising results and is gaining popularity as a predictive biomarker [47]. A recent study reported an association between high TMB (≥175 mutations/exome) and clinically meaningful improvement in the efficacy of pembrolizumab monotherapy in solid tumors [48]. The limitations in the utilization of the PD-1/PD-L1 axis have prompted the investigation of additional checkpoints such as CTLA-4, which downregulates T-cell activation during priming phases in lymphoid organs. While CTLA-4 inhibitors such as ipilimumab have shown limited efficacy as monotherapy in HNSCC, combination strategies with PD-1/PD-L1 inhibitors are under exploration to enhance T-cell activation both at the tumor site and in draining lymph nodes [11,49,50]. Beyond PD-1 and CTLA-4, emerging immune checkpoints are gaining attention in HNSCC. Lymphocyte activation gene-3 (LAG-3), T-cell immunoglobulin and mucin domain-containing protein 3 (TIM-3), and T cell immunoreceptor with Ig and ITIM domains (TIGIT) are highly expressed in exhausted T cells within the TME and contribute to immune dysfunction [49,51,52]. LAG-3, for example, binds MHC class II molecules and synergizes with PD-1 to suppress T-cell function, suggesting that dual blockade may have synergistic anti-tumor effects. TIM-3, through its interactions with galectin-9 and other ligands, further promotes T-cell exhaustion and tolerance [53,54]. TIGIT modulates T-cell and natural killer (NK) cell activity by competing with CD226 for binding to CD155, tipping the balance toward immune suppression [55,56]. Clinical development of inhibitors targeting these emerging checkpoints is advancing quickly. Trials combining PD-1/PD-L1 inhibitors with LAG-3, TIM-3, or TIGIT antagonists are underway in various solid tumors, including HNSCC [57,58]. Early-phase studies indicate that dual checkpoint inhibition may overcome primary or acquired resistance observed with PD-1 monotherapy. In addition to intrinsic checkpoint upregulation, extrinsic factors within the TME—such as chronic inflammation, hypoxia, and metabolic stress—further induce immune checkpoint expression on effector cells and contribute to adaptive resistance mechanisms [59,60]. For instance, hypoxia has been shown to upregulate PD-L1 via hypoxia-inducible factor 1-alpha (HIF-1α)–mediated transcriptional activation, providing a direct link between the metabolic landscape and immune escape in HNSCC [61,62]. Given the complex immune landscape of HNSCC, combination strategies that target multiple checkpoints or concurrently modulate other elements of the TME (e.g., CAFs, hypoxia) are gaining traction as rational therapeutic approaches. Trials combining PD-1/PD-L1 inhibitors with TGF-β inhibitors, VEGF-targeting agents, or metabolic modulators are ongoing and may yield synergistic effects [33,63,64]. Ultimately, a deeper understanding of immune checkpoint biology and resistance mechanisms in HNSCC will guide the development of next-generation immunotherapies, potentially transforming patient outcomes through more effective and durable immune reactivation.

## 4 Cancer-Associated Fibroblasts and Stromal Reprogramming

CAFs are among the most abundant and functionally versatile components of the TME in HNSCC. Unlike normal fibroblasts, which primarily maintain tissue homeostasis and wound healing, CAFs acquire an activated phenotype that profoundly supports tumor progression, immune evasion, and therapeutic resistance [20,65]. Recent evidence from an immunohistochemical study on oral squamous cell carcinoma (OSCC) showed that a higher density of CAFs significantly correlates with advanced T, N, and TNM stages, and is notably associated with local recurrence [66]. CAFs in HNSCC are not a homogeneous population but rather consist of distinct subsets with divergent functions. Two primary phenotypic subtypes have been described: myofibroblastic CAFs (myCAFs) and inflammatory CAFs (iCAFs) [67–69]. myCAFs are characterized by high expression of α-smooth muscle actin (α-SMA) and are primarily involved in ECM remodeling, leading to desmoplasia and increased tissue stiffness. This mechanical remodeling not only creates a physical barrier to immune cell infiltration and drug delivery but also provides pro-survival and pro-invasive signals to tumor cells [23,25,70–72] Moreover, recent comparative studies in bladder cancer have shown that CAFs derived from recurrent tumors exhibit greater potential to induce epithelial-mesenchymal transition (EMT), stemness features, and therapeutic resistance, underscoring their clinical relevance in recurrence and treatment failure [73]. In contrast, iCAFs secrete a wide array of cytokines and chemokines, such as interleukin-6 (IL-6), CXCL12, and interleukin-1 beta (IL-1β), which modulate immune cell recruitment and polarization, promoting an immunosuppressive TME. A central signaling axis in CAF activation and function is the TGF-β pathway, which drives the differentiation of fibroblasts into α-SMA-positive myofibroblasts and stimulates the production of ECM components and immunosuppressive cytokines [26,74]. Elevated TGF-β signaling in HNSCC correlates with poor prognosis, increased metastatic potential, and resistance to ICIs [75,76]. Therefore, targeting TGF-β represents a promising strategy to modulate CAF biology and recondition the TME. IL-6 is another key cytokine produced by CAFs that enhances tumor growth and immune evasion. IL-6 promotes the activation of signal transducer and activator of transcription 3 (STAT3) signaling in both tumor cells and infiltrating immune cells, leading to increased proliferation, survival, and expansion of regulatory immune subsets such as Tregs and MDSCs [71,74]. Elevated IL-6 levels in HNSCC have been associated with worse clinical outcomes and resistance to therapy. The CXCL12/C-X-C chemokine receptor type 4 (CXCR4) axis also plays a crucial role in CAF-mediated immunosuppression and immune exclusion. CXCL12, abundantly secreted by CAFs, creates chemokine gradients that physically segregate effector T cells from the tumor core, thereby impairing anti-tumor immune responses [21,77]. In addition to these chemokine-driven mechanisms, CAFs have also been shown to directly contribute to immune evasion through the expression of immune checkpoint ligands such as PD-L1. While PD-L1 is classically associated with tumor cells and immune infiltrates, its expression on CAFs represents a parallel stromal mechanism of T cell suppression that operates independently of tumor-intrinsic checkpoint pathways [23,25]. Similarly, IL-6 produced by CAFs exerts paracrine effects distinct from tumor-derived IL-6, preferentially activating STAT3 signaling in adjacent immune and epithelial cells. This underscores the unique and non-redundant contribution of stromal elements to the immunosuppressive microenvironment. Pharmacological blockade of CXCR4 has been shown to enhance T-cell infiltration and sensitize tumors to immunotherapy in preclinical models, supporting the rationale for combination strategies targeting this pathway in HNSCC. Given their multifaceted roles, CAFs have emerged as attractive therapeutic targets. Several CAF-targeted strategies are currently under investigation. These include TGF-β inhibitors (e.g., galunisertib), IL-6 neutralizing antibodies (e.g., tocilizumab), and CXCR4 antagonists (e.g., plerixafor) [75,78,79]. Additionally, innovative approaches aim to reprogram CAFs rather than deplete them entirely, as certain CAF subsets may possess tumor-restraining functions. Agents targeting fibroblast activation protein (FAP), or modulating mechanical properties of the ECM, are being explored to render the stroma less permissive to tumor growth and more supportive of immune infiltration [80,81]. Importantly, the dynamic crosstalk between CAFs and other components of the TME—including immune cells, endothelial cells, and tumor cells—creates a complex network of reciprocal interactions. Disrupting these CAF-mediated networks could not only attenuate tumor progression but also enhance the efficacy of existing therapies, particularly ICIs and cytotoxic agents, by overcoming stromal-mediated resistance mechanisms [15,25,82]. In conclusion, CAFs represent a central node in the HNSCC TME that orchestrates tumor supportive inflammation, ECM remodeling, and immunosuppression. Therapeutic strategies aimed at reprogramming or selectively targeting CAFs and their associated signaling pathways hold significant promise for improving clinical outcomes in HNSCC patients.

## 5 Targeting Hypoxia and Metabolic Adaptations

Hypoxia, or reduced oxygen availability, is a prominent feature of the TME in HNSCC and plays a central role in promoting tumor aggressiveness, immune evasion, and therapeutic resistance. HNSCC exhibits a particularly high hypoxic microenvironment compared to other solid tumours [83]. Rapid tumor growth, coupled with aberrant and inefficient angiogenesis, creates hypoxic regions within tumors, which in turn drive profound changes in cellular metabolism and signaling pathways [14,28]. At the molecular level, hypoxia stabilizes HIFs, particularly HIF-1α, which functions as a master regulator of the adaptive response to low oxygen conditions. Under normoxic conditions, HIF-1α is rapidly degraded via the von Hippel-Lindau (VHL) pathway; however, under hypoxia, HIF-1α accumulates and translocates to the nucleus, where it activates the transcription of numerous genes involved in angiogenesis (e.g., VEGF), glycolysis, ECM remodeling, and immune modulation [84,85]. The angiogenic switch induced by HIF-1α leads to the production of structurally and functionally abnormal blood vessels, which paradoxically perpetuate hypoxia rather than resolve it. Hypoxia-driven angiogenesis not only supports tumor growth and metastasis but also creates a physical and functional barrier to effective drug delivery [84,86,87]. In HNSCC, high levels of HIF-1α and associated hypoxia signatures correlate with poor prognosis, increased metastatic potential, and resistance to radiotherapy and chemotherapy [14,88]. In addition to driving angiogenesis, hypoxia profoundly reprograms tumor metabolism. Under hypoxic conditions, tumor cells preferentially shift from oxidative phosphorylation to aerobic glycolysis (the Warburg effect), leading to the production of lactate even in the presence of oxygen [89]. Accumulation of lactate acidifies the TME, which impairs cytotoxic T-cell and NK cell function, promotes Treg development, and facilitates tumor immune escape [31,90,91]. Furthermore, lactate serves as a metabolic fuel for neighboring cancer and stromal cells, promoting a symbiotic metabolic ecosystem that sustains tumor progression. Glutamine metabolism also plays a crucial role in hypoxic adaptation. Tumor cells increase their reliance on glutaminolysis to generate energy and biosynthetic precursors necessary for survival and proliferation under low oxygen conditions [92]. Targeting glutamine metabolism is therefore emerging as a promising therapeutic strategy to disrupt tumor metabolic flexibility. Exosomes, small extracellular vesicles secreted by various cell types, serve as key signaling molecules that regulate numerous physiological and pathological processes, including cancer development. Hypoxia promotes exosome release from cancer cells and can alter their microRNA (miRNA) profiles, contributing to tumor progression [93]. miRNAs from tumor-derived exosomes (miR-192 and miR-215) can target Caveolin-1 and suppress TGF-β/mothers against decapentaplegic homolog (SMAD) signaling, leading to the differentiation of normal fibroblasts into CAFs, while miR-21 does this by targeting YOD1 [94]. Additionally, lysyl oxidase-like 2 (LOXL2)-rich hypoxia-derived exosomes deliver LOXL2 to non-hypoxic HNSCC cells, triggering EMT, enhancing invasion, and promoting pre-metastatic niche formation via the focal adhesion kinase (FAK)/Src pathway [95]. Given the central role of hypoxia and metabolic reprogramming in HNSCC progression, several therapeutic strategies have been developed to target these pathways. One promising approach involves the use of hypoxia-activated prodrugs (HAPs), such as evofosfamide (TH-302). Evofosfamide is selectively activated under hypoxic conditions, releasing a cytotoxic agent that induces DNA cross-linking and tumor cell death [96–98]. Although early-phase trials showed some promise, later-stage studies have yielded mixed results, highlighting the need for better patient selection based on hypoxia biomarkers [85,99]. While their role in HNSCC remains to be fully established, targeting HIFs offers a rational approach to disrupt hypoxia-driven oncogenic signaling. Metabolic inhibitors targeting glycolysis (e.g., 2-deoxy-D-glucose (2-DG)) or lactate dehydrogenase A (LDHA) are also being explored to neutralize the immunosuppressive and pro-tumorigenic effects of lactate accumulation [29,98,100,101]. Additionally, indoleamine 2,3-dioxygenase (IDO) inhibitors, which modulate tryptophan metabolism and immune suppression, have shown potential in early-phase trials, although phase III studies have been less encouraging [102,103]. Combinatorial approaches that integrate hypoxia-targeted therapies with standard treatments such as radiotherapy, chemotherapy, or immunotherapy are actively being pursued. Hypoxia not only contributes to intrinsic resistance to these modalities but also creates a TME that is less accessible to immune effector cells. Therefore, reoxygenating tumors or disrupting hypoxia-adaptive pathways may sensitize tumors to existing treatments and enhance therapeutic efficacy [86,100]. In conclusion, hypoxia and metabolic adaptations are central to the pathophysiology of HNSCC and represent promising therapeutic targets (Table 2). Continued efforts to refine biomarker-driven patient selection and to develop rational combination strategies will be essential to fully exploit the vulnerabilities conferred by tumor hypoxia and metabolic reprogramming.

**Table 2:** Emerging therapeutic targets within the tumor microenvironment (an original table reproduced from the current literature data)

Target	Pathway/Mechanism	Therapeutic strategy
Immune checkpoints	PD-1, PD-L1, CTLA-4, LAG-3, TIM-3, TIGIT	Immune checkpoint inhibitors (monotherapy or combinations) [46,49–52,55,56]
CAF-related signals	TGF-β, CXCL12/CXCR4 axis, IL-6	Stromal reprogramming (TGF-β inhibitors, CXCR4 antagonists, IL-6 blockade) [23,25,74,75,77]
Hypoxia and angiogenesis	HIF-1α, VEGF pathways	Hypoxia-activated prodrugs, anti-angiogenic therapies [84,85,88]
Tumor metabolism	Glycolysis, lactate production, glutaminolysis	Metabolic inhibitors (e.g., LDHA inhibitors, glutaminase inhibitors) [98,100,101]

Note: PD-1, Programmed Death-1; PD-L1, Programmed Death-Ligand 1; CTLA-4, Cytotoxic T-Lymphocyte-Associated Protein 4; LAG-3, Lymphocyte Activation Gene-3; TIM-3, T-cell Immunoglobulin and Mucin Domain-Containing Protein 3; TIGIT, T-cell Immunoreceptor with Ig and ITIM Domains; TGF-β, Transforming Growth Factor Beta; CXCL12, C-X-C Motif Chemokine Ligand 12; CXCR4, C-X-C Chemokine Receptor Type 4; IL-6, Interleukin-6; HIF-1α, Hypoxia-Inducible Factor 1-Alpha; VEGF, Vascular Endothelial Growth Factor; LDHA, Lactate Dehydrogenase A.

## 6 Drug Delivery and Therapeutic Penetration

One of the major challenges in the effective treatment of HNSCC lies in overcoming the physical and functional barriers posed by the TME. Dense ECM deposition, abnormal vasculature, elevated interstitial fluid pressure, and hypoxia collectively restrict the penetration and distribution of therapeutic agents, ultimately reducing treatment efficacy [7,86,104,105]. The dense and rigid ECM, predominantly produced by CAFs, not only hinders the diffusion of chemotherapeutic drugs but also impairs immune cell infiltration [22,26,65,106]. Additionally, abnormal tumor vasculature characterized by leaky, tortuous, and poorly perfused blood vessels leads to heterogeneous drug delivery, resulting in hypoxic and treatment-resistant niches within tumors [86,107,108]. Therefore, innovative strategies aimed at enhancing therapeutic delivery and overcoming TME-mediated barriers are critical to improving clinical outcomes. Nanotechnology-based drug delivery systems have emerged as a promising approach to enhance the selective accumulation of therapeutics in tumors while minimizing systemic toxicity. Nanoparticles, liposomes, and micelles can be engineered to exploit the enhanced permeability and retention (EPR) effect inherent to tumors, thereby facilitating preferential drug delivery to the tumor site [109]. Moreover, nanoparticles can be functionalized with ligands targeting specific tumor or stromal markers, such as epidermal growth factor receptor (EGFR), integrins, or FAP, to further improve targeting specificity. In the context of HNSCC, preclinical studies have demonstrated the utility of liposomal cisplatin formulations, which exhibit improved tumor penetration and reduced nephrotoxicity compared to free cisplatin [110]. Similarly, polymeric nanoparticles delivering paclitaxel or doxorubicin have shown enhanced anti-tumor efficacy by overcoming stromal barriers and achieving sustained drug release. Another innovative strategy involves local drug delivery platforms such as hydrogels, implantable depots, and *in situ*-forming biomaterials. These systems allow for the controlled release of therapeutics directly into the tumor bed or surgical resection margins, thereby achieving high local concentrations while limiting systemic exposure [110–112]. Such approaches are particularly attractive in HNSCC, where anatomical accessibility facilitates local interventions [111,113,114]. Targeted gene silencing through small interfering RNA (siRNA) holds substantial promise in cancer treatment. The utilization of siRNA faces challenges due to its vulnerability to degradation by ribonucleases, limited stability, potential to trigger inflammatory responses, and insufficient targeting specificity. Nanoparticle siRNA-carriers have shown effectiveness in suppressing HNSCC growth by targeting ribonucleotide reductase subunit M2 [113]. Oncolytic virotherapy represents another highly promising method for enhancing therapeutic penetration and immune activation within the TME. Oncolytic viruses (OVs) selectively infect and lyse tumor cells while simultaneously stimulating anti-tumor immunity through the release of tumor-associated antigens and danger signals [105,115–117]. Importantly, OVs can disrupt the tumor stroma, degrade ECM components, and improve immune cell infiltration, thus mitigating the barriers imposed by the TME [15,79,104,105,118]. In HNSCC, several OVs, including modified herpes simplex viruses (e.g., talimogene laherparepvec (T-VEC)) and adenoviruses, have shown promising preclinical and clinical activity, particularly when combined with ICIs. Emerging multi-modal nanoplatforms that integrate chemotherapeutics, immunomodulators, and OVs offer an exciting frontier in HNSCC treatment. These systems can simultaneously remodel the TME, deliver cytotoxic agents, and prime anti-tumor immune responses, providing a comprehensive approach to overcoming therapeutic resistance. Nevertheless, several challenges remain in the field of drug delivery to HNSCC tumors. Heterogeneous TME characteristics, rapid clearance by the reticuloendothelial system (RES), and off-target effects pose significant hurdles. Strategies such as PEGylation (polyethylene glycol coating), active targeting, and stimuli-responsive release (e.g., pH, enzyme, or redox-sensitive systems) are being developed to optimize delivery and biodistribution [105,114,119]. In recent years, nanotechnology-based drug delivery systems have rapidly evolved, offering targeted and multifunctional platforms for overcoming the hostile tumor microenvironment in HNSCC. Table 3 summarizes notable examples of nanodrug delivery strategies evaluated in preclinical and early clinical settings, highlighting their design, therapeutic agents, mechanisms of action, and potential advantages. In conclusion, effective therapeutic delivery in HNSCC requires strategies that not only navigate but actively remodel the hostile TME. Advances in nanotechnology, biomaterials, and oncolytic virotherapy offer promising solutions to enhance drug penetration, immune activation, and overall treatment efficacy. Future research should focus on integrating these approaches into biomarker-driven clinical protocols to maximize patient benefit.

**Table 3:** Recent nanodrug delivery systems evaluated in head and neck squamous cell carcinoma (HNSCC) (an original table reproduced from the current literature data)

Nanocarrier type	Therapeutic agent (s)	Target/ Mechanism	Preclinical/ Clinical	Key outcome (s)	Reference
**Hyaluronan**	Cisplatin	Enhanced accumulation	Preclinical	Improved tumor uptake, reduced nephrotoxicity	[110]
**Polymeric nanoparticles**	Paclitaxel	Intratumoral sustained release, induction of apoptosis, improved tumor targetting	Preclinical	Enhanced inhibitory activities against tumor growth and neovascularization without obvious side effects.	[112]
**Lipid-polymer hybrid NPs**	siRNA (RRM2 gene)	Gene silencing in tumor cells	Preclinical	Inhibited tumor growth *in vivo*	[113]
**PEGylated nanocarriers**	Curcumin	Anti-inflammatory, chemosensitizing effects	Preclinical	Enhanced stability and bioavailability. Sustained drug release.	[114]
**Nanogels/ Hydrogels**	Variable	Local drug depot post-resection	Preclinical	High local concentration, reduced systemic toxicity	[111]
**Oncolytic virus-loaded NPs**	T-VEC, adenovirus	Immune activation + TME modulation	Preclinical/ early clinical	Synergistic anti-tumor activity with ICIs	[105,115]

Note: siRNA, small interfering RNA; RRM2, ribonucleotide reductase subunit M2; PEGylated, polyethylene glycol coating; NPs, nanoparticles; T-VEC, talimogene laherparepvec; TME, tumor microenvironment; ICIs, immune checkpoint inhibitors.

## 7 Clinical Trials and Translational Perspectives

The recognition of the TME as a key driver of therapy resistance and disease progression in head and HNSCC has led to a surge in clinical trials aiming to modulate TME components to improve patient outcomes (Table 4). These efforts focus on combining ICIs, stromal targeting agents, hypoxia modulators, and novel delivery systems, often within biomarker-driven frameworks [5,34,57,120]. One major avenue of investigation involves combination immunotherapy trials. Building on the success of PD-1/PD-L1 blockade, numerous clinical studies are testing combinations of checkpoint inhibitors with agents targeting additional immune checkpoints such as LAG-3, TIM-3, and TIGIT. For example, the RELATIVITY-047 trial evaluating nivolumab plus the LAG-3 inhibitor relatlimab has shown promise in melanoma, and similar combinations are now being explored in HNSCC [12,121]. Additionally, trials combining checkpoint blockade with TGF-β inhibitors (e.g., bintrafusp alfa) seek to overcome immune exclusion mediated by stromal and fibrotic barriers [122,123]. Biomarker-driven trials that select patients based on hypoxia signatures (e.g., HIF-1α) expression, hypoxia gene signatures) are underway to better identify those who may benefit from such approaches. Oncolytic virotherapy, as reviewed recently [15,79,104,105,116–118], represents another frontier in translational HNSCC research. T-VEC, a genetically modified herpes simplex virus encoding granulocyte-macrophage colony-stimulating factor (GM-CSF), has demonstrated durable responses in melanoma and is now being tested in HNSCC, particularly in combination with ICIs [124]. Other oncolytic platforms, including adenoviruses and reoviruses, are similarly being explored. Moreover, nanoparticle-based delivery systems and local drug release strategies are entering clinical development, aiming to improve the penetration and retention of chemotherapeutic agents and immunotherapies within the hostile TME. Several trials are assessing nanoparticle formulations of cisplatin, paclitaxel, and immune adjuvants in patients with advanced HNSCC [113,125]. A critical advancement in translational research is the move toward biomarker-driven patient selection. Recognizing the heterogeneity of the TME, contemporary trials increasingly incorporate molecular and immune profiling to stratify patients. Biomarkers such as PD-L1 expression, TMB, immune gene signatures, hypoxia markers, and CAF-related gene expression are being explored to guide therapy allocation [126]. Future opportunities in drug development focus on rational combination therapies, early intervention in the adjuvant setting, and the integration of emerging modalities such as adoptive cell therapies and personalized cancer vaccines tailored to individual TME characteristics. In parallel, improvements in imaging and non-invasive monitoring, such as radiomics and liquid biopsies, will facilitate real-time assessment of TME modulation and treatment response [9,127].

**Table 4:** Ongoing and completed clinical trials targeting the TME in HNSCC

NCT number	Intervention	Study status	Phase
NCT01848834	Pembrolizumab	Completed	Phase I
NCT02105636	Nivolumab	Completed	Phase III
	Cetuximab		
	Methotrexate		
	Docetaxel		
NCT02358031	Pembrolizumab	Completed	Phase III
	Cisplatin		
	Carboplatin		
	5-FU		
	Cetuximab		
NCT02643550	Monalizumab	Completed	PhaseI|PhaseII
	Cetuximab		
	Anti-PD(L)1		
NCT03065062	Palbociclib	Recruiting	Phase I
	Gedatolisib		
NCT03589339	NBTXR3	Recruiting	Phase I
	SABR		
	Nivolumab Pembrolizumab		
NCT03739931	mRNA-2752	Active (not recruiting)	Phase I
	Durvalumab		
NCT04080804	Nivolumab	Recruiting	Phase II
	Relatlimab		
	Ipilimumab		
NCT04811027	Eftilagimod alpha	Active (not recruiting)	Phase II
	Pembrolizumab		
NCT04862455	Hafnium Oxide-containing Nanoparticles NBTXR3	Active (not recruiting)	Phase II
	Hypofractionated Radiation Therapy		
	Pembrolizumab		
	Stereotactic Body Radiation Therapy		
NCT04892173	JNJ-90301900 (NBTXR3)	Recruiting	Phase III
	Cetuximab		
	Radiation therapy		
NCT05287113	Retifanlimab	Active (not recruiting)	Phase II
	INCAGN02385		
	Placebo		
NCT05366166	Pembrolizumab	Recruiting	Phase II
	Olaparib		
	Cisplatin		
	IMRT		

Note: 5-FU, 5-fluorouracil; PD(L)1, programmed death ligand 1; SABR, stereotactic ablative radiotherapy; mRNA, messenger RNA; IMRT, intensity modulated radiation therapy.

Despite promising early-phase signals, most clinical trials targeting the tumor microenvironment (TME) in HNSCC have failed to translate immune activation or local tumor control into meaningful survival benefits. Key limitations include insufficient patient selection due to a lack of validated biomarkers, difficulty overcoming TME-mediated immunosuppression, and toxicity or limited efficacy of combination regimens. These challenges highlight the complexity of reprogramming the TME and the need for more precise, stratified approaches.

**Figure 3:**
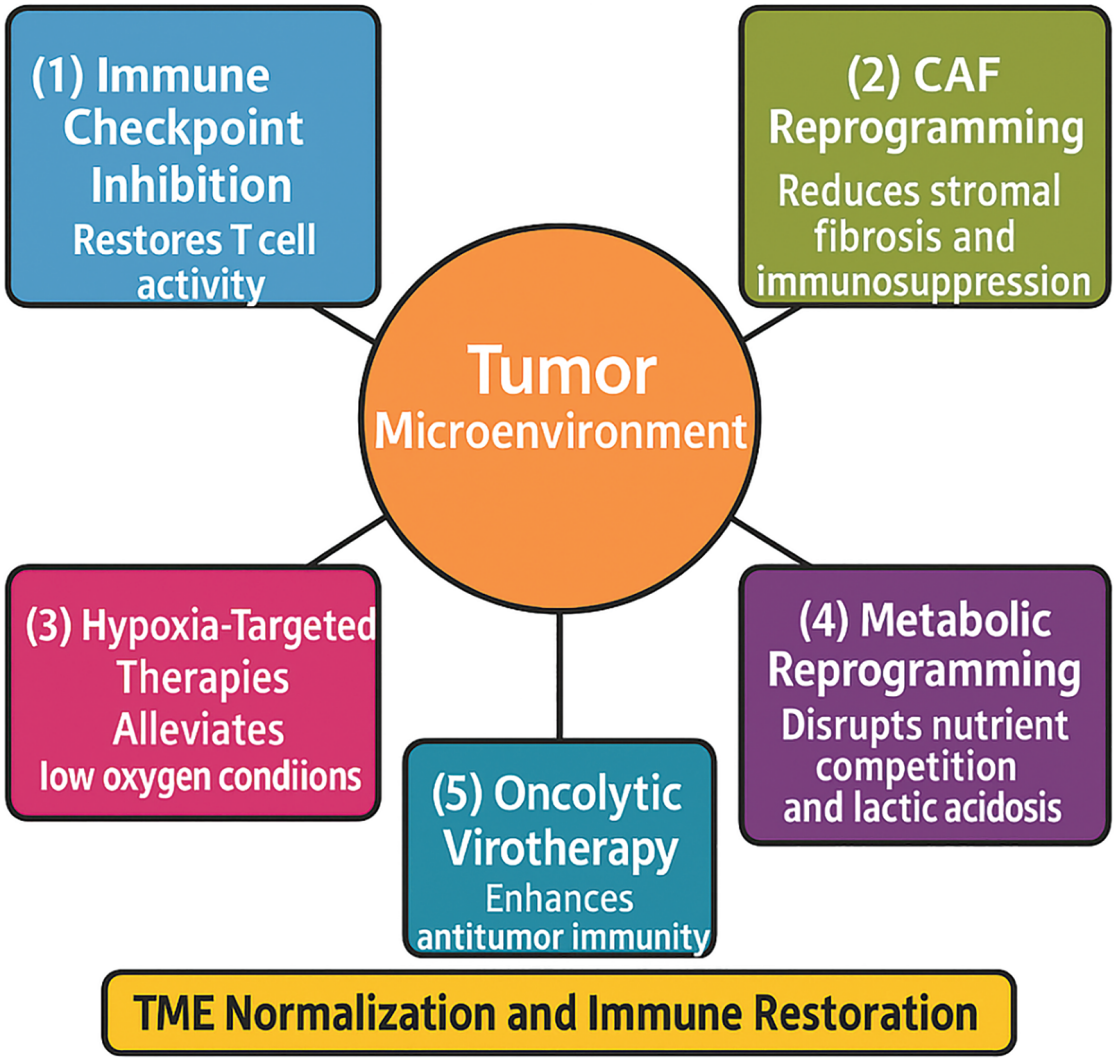
Strategies for therapeutic reprogramming of the tumor microenvironment. The tumor and its surrounding microenvironment impose multiple barriers to effective treatment, including immune suppression, metabolic dysregulation, stromal resistance, and hypoxia. Arrows illustrate key strategies aimed at reprogramming the tumor microenvironment (TME): (**1**) Immune checkpoint inhibition to restore T cell activity by targeting pathways such as programmed cell death protein 1/Programmed Death-Ligand 1 (PD-1/PD-L1) and Cytotoxic T-Lymphocyte-Associated Protein 4 (CTLA-4); (**2**) CAF reprogramming targeting cancer-associated fibroblasts (CAFs) to reduce stromal fibrosis and immunosuppression; (**3**) Hypoxia-targeted therapies that alleviate low oxygen conditions and improve immune cell infiltration; (**4**) Metabolic reprogramming of tumor and stromal cells to disrupt nutrient competition and lactic acidosis; and (**5**) Oncolytic virotherapy, the use of viruses that selectively replicate in tumor cells and enhance antitumor immunity. The central therapeutic goal of these interventions is TME normalization and immune restoration, ultimately improving response to conventional and immunotherapeutic treatments in head and neck squamous cell carcinoma (HNSCC) (Original scheme based on current literature data created using BioRender.com, BioRender, Toronto, ON, Canada)

In conclusion, the landscape of clinical research in HNSCC is rapidly evolving toward TME-centric and precision oncology paradigms. Continued innovation in trial design, including adaptive platforms, biomarker integration, and multidisciplinary collaboration, will be essential to translate these scientific advances into meaningful improvements in survival and quality of life for patients with HNSCC (Fig. 3).

## 8 Combination Therapy and Drug Repurposing Strategies

Combining conventional and immunotherapeutic strategies with repurposed non-oncology drugs has emerged as a promising approach to overcome therapeutic resistance and exploit the vulnerabilities of the tumor microenvironment (TME) in head and neck squamous cell carcinoma (HNSCC). Drug repurposing involves the use of existing medications—originally developed for non-cancer indications—with known safety profiles, to target cancer-related pathways including immunosuppression, angiogenesis, metabolism, and DNA damage repair [128,129].

In this context, several drug classes—ranging from metabolic and anti-inflammatory agents to cardiovascular and neuroactive compounds—have demonstrated preclinical efficacy in modulating the immunosuppressive TME and sensitizing HNSCC tumors to standard therapies [130].

This growing body of evidence underscores the potential of repurposed drugs as cost-effective and biologically versatile components of combination strategies in HNSCC.

### 8.1 Antidiabetic Agents (e.g., Metformin)

Metformin, a widely prescribed antidiabetic agent, exerts pleiotropic anti-cancer effects. Mechanistically, it inhibits mitochondrial complex I and activates AMP-activated protein kinase (AMPK), resulting in mTOR pathway suppression. In HNSCC models, metformin has demonstrated the ability to reduce tumor cell proliferation, cancer stem cell frequency, and enhance radiosensitivity. Its effects on tumor hypoxia and immunometabolism make it a rational candidate for integration with radiotherapy and immune checkpoint inhibitors (ICIs) [131,132].

### 8.2 Anti-Inflammatory Agents (e.g., NSAIDs)

Non-steroidal anti-inflammatory drugs (NSAIDs), particularly selective COX-2 inhibitors like celecoxib, have shown the ability to reverse inflammation-driven immunosuppression within the TME. By inhibiting prostaglandin E2 (PGE2) synthesis, NSAIDs reduce regulatory T cell (Treg) infiltration and restore cytotoxic T cell activity. Their synergy with ICIs and chemoradiotherapy is under exploration, with preclinical studies suggesting enhanced antitumor immunity [133,134].

### 8.3 Adrenergic Modulators (e.g., Beta-Blockers)

Beta-adrenergic signaling has been implicated in tumor progression, immune escape, and angiogenesis. Beta-blockers such as propranolol block β-adrenergic receptors and have demonstrated tumor-suppressive effects across various malignancies, including HNSCC. Their immunomodulatory capacity and ability to reduce VEGF-mediated angiogenesis present compelling rationale for inclusion in multimodal protocols [135,136].

### 8.4 Antialcoholism Agents (e.g., Disulfiram)

Disulfiram, used clinically for alcohol aversion, inhibits aldehyde dehydrogenase (ALDH), a key enzyme in cancer stem cell maintenance. It also induces oxidative stress and proteasomal inhibition in tumor cells. When combined with cisplatin or ICIs, disulfiram enhances cytotoxicity and may overcome chemoresistance in HNSCC preclinical models [137,138].

### 8.5 Cardiometabolic Drugs (e.g., Statins, ARBs)

Statins such as simvastatin and atorvastatin inhibit 3-Hydroxy-3-Methylglutaryl Coenzyme A (HMG-CoA) reductase and exert anti-proliferative and pro-apoptotic effects on cancer cells. They may also interfere with CAF activation and reduce ECM rigidity. Angiotensin receptor blockers (ARBs), including losartan, have been shown to normalize tumor vasculature and enhance drug delivery. These agents may mitigate stromal desmoplasia and TME-associated therapy resistance [139,140].

### 8.6 Clinical Integration and Future Prospects

The integration of repurposed drugs into combination regimens for HNSCC is gaining momentum. Ongoing trials are evaluating metformin, NSAIDs, and statins as adjuvants to ICIs or radiotherapy. These agents offer several advantages: well-characterized pharmacokinetics, established safety profiles, affordability, and the potential to target multiple TME pathways simultaneously. However, challenges remain regarding optimal dosing, sequencing, and biomarker-driven patient selection. Future research should emphasize rational combination design and clinical validation in biomarker-stratified populations.

## 9 Conclusion and Future Directions

Head and neck squamous cell carcinoma (HNSCC) continues to pose formidable therapeutic challenges due to its biological complexity, treatment resistance, and high recurrence rates. It is increasingly evident that the tumor microenvironment (TME) is not merely a bystander but a central orchestrator of cancer progression, immune evasion, and therapeutic failure. Recent advances have uncovered the intricate interplay between malignant cells and various TME components, including immunosuppressive cells, cancer-associated fibroblasts (CAFs), hypoxia-induced metabolic pathways, and extracellular matrix remodeling.

Targeting these components offers a promising avenue to overcome the limitations of conventional therapies and reinvigorate anti-tumor immunity. Therapeutic strategies include immune checkpoint blockade, stromal reprogramming, hypoxia-targeted therapies, metabolic inhibitors, and innovative drug delivery platforms. Particularly promising approaches involve the use of oncolytic viruses and nanotechnology-based systems, both of which have shown encouraging results in preclinical and early clinical studies.

However, several barriers hinder the clinical translation of these strategies. One critical challenge is the lack of validated and reliable biomarkers for patient selection and therapy monitoring. In addition, CAF heterogeneity and the dynamic remodeling of the TME in response to treatment complicate the reproducibility of preclinical findings and limit their relevance in human trials. The economic burden and ethical considerations associated with personalized approaches, such as oncolytic virotherapy, engineered nanocarriers, and combination immunotherapy, must also be addressed. These concerns are especially pressing in resource-limited settings.

Future directions should prioritize biomarker-driven personalized therapies based on each patient’s immune, stromal, and metabolic profiles. The integration of spatial transcriptomics, single-cell sequencing, and advanced imaging technologies will enable precise characterization of the TME and real-time assessment of treatment response. Rational combination regimens that simultaneously target multiple aspects of the TME may yield synergistic effects and help prevent resistance. Achieving these goals will require multidisciplinary collaboration and adaptive clinical trial designs that include meaningful translational endpoints.

In conclusion, reprogramming the TME in HNSCC represents a transformative shift from tumor-centric to ecosystem-centric oncology. By dismantling the protective barriers created by the TME and exploiting its vulnerabilities, it may be possible to deliver personalized, durable, and potentially curative therapies for patients with HNSCC.

## Data Availability

Not applicable.

## Abbreviations

2-DG: 2-Deoxy-D-Glucose
α-SMA: Alpha-Smooth Muscle Actin
AMPK: AMP-Activated Protein Kinase
ARB: Angiotensin Receptor Blocker
CAFs: Cancer-Associated Fibroblasts
CTL: Cytotoxic T Lymphocyte
CTLA-4: Cytotoxic T-Lymphocyte-Associated Protein 4
CXCL12: C-X-C Motif Chemokine Ligand 12
CXCR4: C-X-C Chemokine Receptor Type 4
ECM: Extracellular Matrix
EGFR: Epidermal Growth Factor Receptor
EMT: Epithelial-Mesenchymal Transition
EPR: Enhanced Permeability and Retention
FAK: Focal Adhesion Kinase
FAP: Fibroblast Activation Protein
FOXP3: Forkhead Box P3
GM-CSF: Granulocyte-Macrophage Colony-Stimulating Factor
HAPs: Hypoxia-Activated Prodrugs
HIF-1α: Hypoxia-Inducible Factor 1-Alpha
HIFs: Hypoxia-Inducible Factors
HMG-CoA: 3-Hydroxy-3-Methylglutaryl Coenzyme A
HNSCC: Head and Neck Squamous Cell Carcinoma
ICIs: Immune Checkpoint Inhibitors
IDO: Indoleamine 2,3-Dioxygenase
iCAFs: Inflammatory Cancer-Associated Fibroblasts
IL-1β: Interleukin-1 Beta
IL-6: Interleukin-6
IL-10: Interleukin-10
IMRT: Intensity Modulated Radiation Therapy
LAG-3: Lymphocyte Activation Gene-3
LDHA: Lactate Dehydrogenase A
LOXL2: Lysyl Oxidase-Like 2
MDSCs: Myeloid-Derived Suppressor Cells
miRNA: MicroRNA
myCAFs: Myofibroblastic Cancer-Associated Fibroblasts
NK: Natural Killer
NSAIDs: Non-Steroidal Anti-Inflammatory Drugs
ORRs: Overall Response Rates
OVs: Oncolytic Viruses
PD-1: Programmed Death-1
PD-L1: Programmed Death-Ligand 1
PEGylation: Polyethylene Glycol Coating
PGE2: Prostaglandin E2
PT2385/PT2977: HIF-1α Transcriptional Inhibitors (incl. Belzutifan)
RES: Reticuloendothelial System
SABR: Stereotactic Ablative Radiotherapy
siRNA: Small Interfering RNA
SMAD: Mothers Against Decapentaplegic Homolog
STAT3: Signal Transducer and Activator of Transcription 3
T-VEC: Talimogene Laherparepvec
TAM: Tumor-Associated Macrophage
TCR: T-Cell Receptor
TGF-β: Transforming Growth Factor Beta
TIGIT: T-Cell Immunoreceptor with Ig and ITIM Domains
TILs: Tumor-Infiltrating Lymphocytes
TIM-3: T-Cell Immunoglobulin and Mucin Domain-Containing Protein 3
TMB: Tumor Mutational Burden
TME: Tumor Microenvironment
Tregs: Regulatory T Cells
VEGF: Vascular Endothelial Growth Factor
VHL: Von Hippel–Lindau
